# Inhibition of glycolysis represses the growth and alleviates the endoplasmic reticulum stress of breast cancer cells by regulating TMTC3

**DOI:** 10.1515/med-2023-0635

**Published:** 2023-04-10

**Authors:** Xue Hu, Baoliang Guo, Tong Sun, Wan Wang

**Affiliations:** Department of Breast Surgery, China-Japan Union Hospital of Jilin University, Changchun City, Jilin Province, 130033, China; Department of General Surgery, The Second Affiliated Hospital of Harbin Medical University, Harbin City, Heilongjiang Province, China; Department of Breast Surgery, China-Japan Union Hospital of Jilin University, No. 126 Xiantai Avenue, Nanguan District, Changchun City, Jilin Province, 130033, China

**Keywords:** breast cancer, Warburg effect, glycolysis, endoplasmic reticulum stress, transmembrane *O*-mannosyltransferase-targeting cadherins 3

## Abstract

Considering the role of glycolysis inhibition as a novel therapeutic strategy for cancer, including breast cancer (BC), we wondered whether glycolysis could affect BC progression by regulating transmembrane *O*-mannosyltransferase-targeting cadherins 3 (TMTC3). Following the intervention, lactic acid production in BC cells was monitored, and viability, proliferation, and apoptosis assays were performed. The expressions of TMTC3 and endoplasmic reticulum (ER) stress- and apoptosis-related factors Caspase-12, C/EBP homologous protein (CHOP), glucose-regulated protein 78 (GRP78), B-cell lymphoma-2 (Bcl-2), and Bcl-2 associated X (Bax) were quantified. TMTC3 was lowly expressed in BC tissue and cell. The promotion of glycolysis via glucose represses TMTC3 expression and apoptosis yet enhances lactic acid production and growth of BC cell, along with promoted levels of Caspase-12, CHOP, GRP78, and Bcl-2 yet repressed level of Bax, while the contrary results were evidenced after 2-deoxyglycouse intervention. Overexpressed TMTC3 additionally abrogated the effects of glycolysis on increasing the viability and proliferation yet inhibiting the apoptosis of BC cells, with the increased expressions of Caspase-12, CHOP, and GRP78, and Bcl-2 yet decreased level of Bax. Collectively, inhibiting glycolysis restrained the growth and attenuated the ER stress of BC cell by regulating TMTC3.

## 1 Introduction

As a heterogenous disease where both genetic and environmental factors are involved, breast cancer (BC), currently, is the leading cause of cancer-associated burden for women along with a continuing increase in the incidence, despite the progression of and devotion to laboratory, epidemiological, and clinical research studies [1,2]. At present, therapeutic strategies for BC mainly include surgical operation, chemotherapy, endocrinotherapy, and molecular-targeted therapy [3]. However, some undesired effects have been demonstrated to emerge as well with the development of resistance to drug and radiation [4]. In addition, due to the technical challenges, the clinical practice of gene therapy is still at its infancy [5]. Therefore, further viable therapeutic options are urgently required for the treatment of BC in clinical practice.

Cancer cells have been proposed to have a different metabolism compared with that of normal cells from which they are derived, and the elevated metabolism has allowed them to sustain higher proliferative rate and resist the cell death signals, as indicated by the capability of utilizing glycolysis as their primary metabolic mode, even in the presence of sufficient oxygen [6]. Such phenomenon is termed as Warburg effect or aerobic glycolysis. Aerobic glycolysis is a crucial metabolic adaptation of cancer cells. Frequently witnessed in cancers, aerobic glycolysis is one of the earliest known evidence related to the metabolic alteration in the neoplasms (including BC) and has been scientifically recognized as a hallmark for the metabolism in the cancer cells, the targeting of which may be contributory to providing therapeutic strategies for cancer [7,8,9]. In addition, aberrant glycolysis is commonly associated with drug resistance in cancer treatment; therefore, targeting glycolysis may be a novel strategy to develop new drugs to benefit patients with drug resistance [10,11].

Aerobic glycolysis can regulate BC proliferation and cell survival. Tang et al. reported that glycolysis-related genes (PRKACB, STMN1, and ZNF292) might provide an effective prognostic predictor for individualized management of BC patients [12]. In BC, transcription factor SIX1 directly increases the expression of many glycolytic genes, promoting the Warburg effect and tumor growth *in vitro* and *in vivo* [9]. Betulinic acid suppresses BC metastasis by targeting GRP78-mediated glycolysis and endoplasmic reticulum (ER) stress apoptotic pathway [13]. Targeting the function of the glucose metabolism might be a promising therapeutic strategy for BC. Nevertheless, the intrinsic mechanism concerning the effects of aerobic glycolysis in BC remains inadequately understood, making it critical to further identify the initial oncogenic signaling so as to develop some viable strategies for the inhibition of glycolysis [14].

Transmembrane *O*-mannosyltransferase-targeting cadherins 3 (TMTC3) is a member of four ER transmembrane *O*-mannosyltransferases, each of which is characterized by the existence of four repeats in the tetratricopeptides [15]. Recent discoveries have unveiled that TMTC3 contributes to the *O*-mannosylation of E-cadherin, the cellular adherence, and the embryonic gastrulation [16]. Dysregulation of glycosylation is one of the important mechanisms leading to tumor heterogeneity [17]. The abnormal expression of glycosyltransferase and its glycan structure are the common features of the occurrence, development, and metastasis of malignant tumors [18]. ER stress is involved in the processes of cellular interactions with the tumor microenvironment, affecting tumor malignant growth, angiogenesis, and progression [19,20,21]. In addition, the participation of TMTC3 in ER stress response has been pointed out as well, together with the modulation on the activity of proteasome and the expression of transcript X-box binding protein 1 [22]. What makes us curious is the discovery addressing the implication of TMTC3 in BC [23].

Based on the above information, we wondered whether glycolysis could affect BC progression by regulating TMTC3. In this study, we explored the interaction between the glycolysis and TMTC3 in BC, the results of which are reported as follows.

## 2 Materials and methods

### 2.1 Bioinformatics analysis

In order to sort the candidate gene for our study, we downloaded the data from the datasets GSE110960 (effect of restricted glycolysis on gene expression in MCF-7 cells) and GSE111204 (effect of restricted glycolysis on gene expression in MDA-MB-231 cells) from Gene Expression Omnibus (https://www.ncbi.nlm.nih.gov/geo/).

The common differentially expressed genes (DEGs) were sorted using GEO2R (https://www.ncbi.nlm.nih.gov/geo/geo2r/), and a Venn diagram was subsequently drawn to identify the common DEGs using Venny online software (version 2.1.0, http://bioinfogp.cnb.csic.es/tools/venny/).

### 2.2 Clinical sample collection

To determine the role of TMTC3 played in BC, 30 paired cancer (defined as “Tumor”) and adjacent tissues (defined as “Normal”) were collected from the Department of Pathology in China−Japan Union Hospital of Jilin University, all of which were harvested from patients admitted from February 2019 to January 2020. All tissues collected were rinsed with saline (IN9000, Solarbio Lifesciences, China) and snap frozen in liquid nitrogen as appropriate.

### 2.3 Cell culture

All cell lines and the materials used for cell culture were ordered from Procell (Wuhan, China, https://www.procell.com.cn/) unless specified otherwise. Human breast epithelial cell MCF-10A (CL-0525) and BC cell lines MCF-7 (CL-0149), MDA-MB-231 (CL-0150), MDA-MB-436 (CL-0383), and SK-BR-3 (CL-0211) were ordered and cultured as needed.

MCF-10A cells were maintained in Dulbecco’s modified Eagle’s medium/F12 medium (PM150312) blended with 5% horse serum (164215), 20 ng/mL of epidermal growth factor (P00033, Solarbio Lifesciences, China), 0.5 μg/mL of hydrocortisone (G8450, Solarbio Lifesciences, China), 10 μg/mL of insulin (I8830, Solarbio Lifesciences, China), 1% non-essential amino acid (PB180424), and 1% penicillin−streptomycin (PB180120). MCF-7 cell was cultured in minimum essential medium (PM150140) with 0.01 mg/mL of insulin. Leibovitz’s L-15 medium (PM151010) was used to ensure the growth of MDA-MB-231 and MDA-MB-436 cells. SK-BR-3 cell was grown in McCoy’s 5A medium (PM150710). All media for BC cells were supplemented with 10% fetal bovine serum (164210) and 1% penicillin−streptomycin.

All cells were finally incubated in the Sanyo MCO-18AIC(UV) CO_2_ incubator (SA-MC018, Marshall Scientific, LLC., Hampton, NH, USA) at 37°C and 5% CO_2_. As TMTC3 was lowly expressed in MCF-7 and MDA-MB-231 cells, these two cells were used for subsequent studies.

### 2.4 Cell treatment and transfection

For cell treatment, both MCF-7 and MDA-MB-231 cells were cultured in the following manner: cells in the control group were cultured normally with no supplementation, while those in the glucose and 2-deoxyglucose groups were cultured in the medium with the supplementation of glucose (10 mM, G8150, Solarbio Lifesciences, China) and 2-deoxyglucose (50 mM, D8930, Solarbio Lifesciences, China), respectively [24].

For transfection, TMTC3 overexpression plasmid was constructed based on the vector pcDNA 3.1 (V790-20, Invitrogen, Carlsbad, CA, USA), and the empty pcDNA 3.1 was used as the negative control (NC).

BC cells MCF-7 and MDA-MB-231 (1 × 10^6^ cells/well) were first seeded in a six-well plate with complete medium at 37°C and 5% CO_2_ to be 90% confluent at the time point of transfection. Then, the transfection on both MCF-7 and MDA-MB-231 cells were successfully performed with the help of lipofectamine 2000 transfection reagent (11668-030, Invitrogen, USA) as per the protocols of the manufacturer. After 48 h, all cells were harvested for subsequent studies.

### 2.5 Lactic acid production determination assay

All procedures of lactic acid production assay were repeated as illustrated previously [25]. The concentration of lactic acid in the harvested lysates was determined using a lactate assay kit (D799851, Sangon Biotech, Shanghai, China) in accordance with the protocols of the manufacturer. MCF-7 and MDA-MB-231 cells (5 × 10^5^ cells/well) with or without transfection were cultured in six-well plates and resuspended in extraction buffer I, followed by lysis at a power of 300 W for 3  min in total (3  s of ultrasonication, at an interval of 7 s) using an XC-II D ultrasonic cell crusher (Nanjing Ningkai Instrument Co., Ltd, Nanjing, China). The lysates were harvested after centrifugation at 12,000 × *g* at 4°C for 10 min in a 5810 Centrifuge (Eppendorf, Hamburg, Germany). A volume of 0.8 mL of the supernatant was collected and added with 0.15 mL of extraction buffer II, followed by another centrifugation at 12,000 × *g* at 4°C for 10 min. The absorbance at 570 nm was monitored by Varioskan^™^ LUX multimode microplate reader (VLBLATD2; ThermoFisher Scientific, Waltham, MA, USA).

### 2.6 Cell-Counting Kit-8 (CCK-8) assay

MCF-7 and MDA-MB-231 cells (2 × 10^3^ cells/well) with different intervention were cultured within 96-well plates at 37°C and 5% CO_2_ for 24 and 48 h, and 10 μL of CCK-8 solution (CA1210; Solarbio Lifesciences, China) was added into each well of the plates for a further 4-h incubation in the incubator. The optical density (OD) value was measured using Varioskan^™^ LUX multimode microplate reader at an absorbance of 450 nm.

Then, the cell viability was calculated using the following formula:

{\rm{Cell\; viability}}( \% )=\frac{{{\rm{OD}}}_{{\rm{dosage}}}-{{\rm{OD}}}_{{\rm{blank}}}}{{{\rm{OD}}}_{{\rm{no\; dosage}}}-{{\rm{OD}}}_{{\rm{blank}}}}\times 100 \% .]

The OD_dosage_ represented the OD value of the wells containing transfected and treated cells and CCK-8 reagent, OD_blank_ symbolized the OD value of the wells with the medium and CCK-8 reagent only, and OD_no dosage_ was the OD value of the wells to which untransfected or untreated cells were added.

### 2.7 Colony formation assay

Following the intervention as needed, MCF-7 and MDA-MB-231 cells (1 × 10^3^ cells/well) were seeded in six-well plates at 37°C and 5% CO_2_ for 14 days, and the colonies formed were fixed with 4% paraformaldehyde (P1110; Solarbio Lifesciences, China) for 15 min and stained with crystal violet (C8470; Solarbio Lifesciences, China) for 30 min. All colonies formed were then observed and photographed using a digital camera (OM-D E-M5 Mark III; Olympus, Tokyo, Japan). The colony formation rates of cells in each group were calculated by the software SigmaPlot 12.0 (Systat Software, Inc., San Jose, CA, USA).

### 2.8 Flow cytometry assay

We collected 1 × 10^6^ transfected MCF-7 and MDA-MB-231 cells, washed with phosphate buffered saline (P1010; Solarbio Lifesciences, China) and suspended using 1 mL of binding buffer (1×), followed by centrifugation at 300 × *g* in a 5810 Centrifuge. The supernatant was abandoned subsequently. All cells were then resuspended with 1 mL of binding buffer (1×), and the density was adjusted to 1 × 10^6^ cells/mL. A volume of 100 μL of cells suspension was added into each tube, and 5 μL of Annexin V-FITC was also added. A gentle blend was performed at room temperature (RT) for 5 min avoiding light, after which 5 μL of propidium iodide (PI) was added into the tube and all cells were additionally incubated at RT for 5 min in the dark. The apoptosis was detected using an Annexin V-FITC/PI apoptosis detection kit (CA1020, Solarbio Lifesciences, China) using a S1000EON flow cytometer (Stratedigm, Inc., San Jose, CA, USA), and all data were analyzed using Kaluza C Analysis Software 1.12 (Beckman Coulter, Indianapolis, IN, USA).

### 2.9 RNA isolation and reverse-transcription quantitative PCR

Total RNA in tissues (both tumor and normal) and cells (BC cells and human breast epithelial cell MCF-10A) was isolated using a total RNA extraction reagent (R1100; Solarbio Lifesciences, China) as guided by the manufacturer, and all isolated RNA samples were preserved in −80°C. The measurement on the isolated RNA was conducted in the NanoDrop^™^ lite spectrophotometer (ND-LITE; ThermoFisher Scientific, USA). All procedures of PCR were then performed by a one-Step RT-PCR kit (T2210; Solarbio Lifesciences, China) and operated in CFX384 Touch real-time PCR system (Bio-Rad, Hercules, CA, USA) under the following detailed conditions: 50°C for 20 min for reverse transcription, 95°C for 3 min for pre-denaturation, followed by 40 cycles of 95°C for 15 s for denaturation, and 60°C for 25 s for final extension.

Relative expressions were quantified using the 2^−ΔΔCT^ method, with glyceraldehyde-3-phosphate dehydrogenase (GAPDH) as the internal reference [26]. The sequences of primers are listed in Table 1.

**Table 1:** Primer for quantitative real-time PCR

Gene	Forward primer	Reverse primer
TMTC3	ATAGTAGGTGTGGTTACTGC	AATACTGTTAAGGGACGGTA
GAPDH	CCTCAACTACATGGTTTACA	TGTTGTCATACTTCTCATGG

### 2.10 Western blot

Relative protein expression was calculated using western blot as confirmed in a previous study [27]. All materials used were the products of Elabsciences (Wuhan, China) unless specified. Total protein in transfected MCF-7 and MDA-MB-231 cells was lysed and extracted using radio-immunoprecipitation assay lysis buffer (E-BC-R327), and the concentration was quantified using bicinchoninic acid (BCA) method with a BCA protein kit (E-BC-K318). A volume of 20 μL of protein lysates was electrophoresed with sodium dodecyl sulfate-polyacrylamide gel electrophoresis (E-IR-R305) and quickly transferred into polyvinylidene fluoride membrane (E-BC-R266). Then the membrane was blocked by 5% skimmed milk at RT for 2 h and incubated in primary and secondary antibodies (the condition for the incubation with the primary antibodies was 4°C overnight and that for secondary antibodies was RT for 1 h).

For subsequent procedures of visualization, the membrane was rinsed using tris-buffer saline tween (E-BC-R335) three times, and was visualized using an enhanced chemiluminescent substrate detection kit (E-BC-R347) within an imaging system (Tanon 5200CE, Tanon, Shanghai, China), and gray values of each band were calculated by ImageJ 5.0 (Bio-Rad, USA).

The primary antibodies (Abcam, Cambridge, UK) used here were those against Caspase-12 (ab62484, 42 kDa), C/EBP homologous protein (CHOP, ab11419, 31 kDa), glucose-regulated protein 78 (GRP78, ab21685, 75 kDa), B-cell lymphoma-2 (Bcl-2, ab182858, 26 kDa), Bcl-2 associated X (Bax, ab32503, 21 kDa), and housekeeping control GAPDH (ab8245, 36 kDa), and the secondary antibodies were the horseradish peroxidase (HRP)-conjugated goat anti-rabbit IgG (E-AB-1003) and HRP-conjugated goat anti-mouse IgG (E-AB-1001). All antibodies (both primary and secondary) were diluted to a ratio of 1:2,000 for our current study.

### 2.11 Statistical analyses

All statistical analyses were implemented with Graphpad Prism 8.0 (GraphPad, Inc., La Jolla, CA, USA), where all data were the indication of three independent tests and expressed as mean ± standard deviation (SD). Statistical significance, which was determined with one-way analysis of variance followed by Bonferroni *post hoc* test and paired *t* test as appropriate, was defined when *p-*value was lower than 0.05.


**Ethics statement:** The ethics committee of China−Japan Union Hospital of Jilin University has carefully reviewed and approved the conduction of our study (endorse number: 2021-KYLL-030008). Meanwhile, all recruited patients have signed the informed consent in written form and agreed to the usage of their tissues in our study.

## 3 Results

### 3.1 TMTC3 was lowly expressed in BC tissue and cell

**Figure 1:**
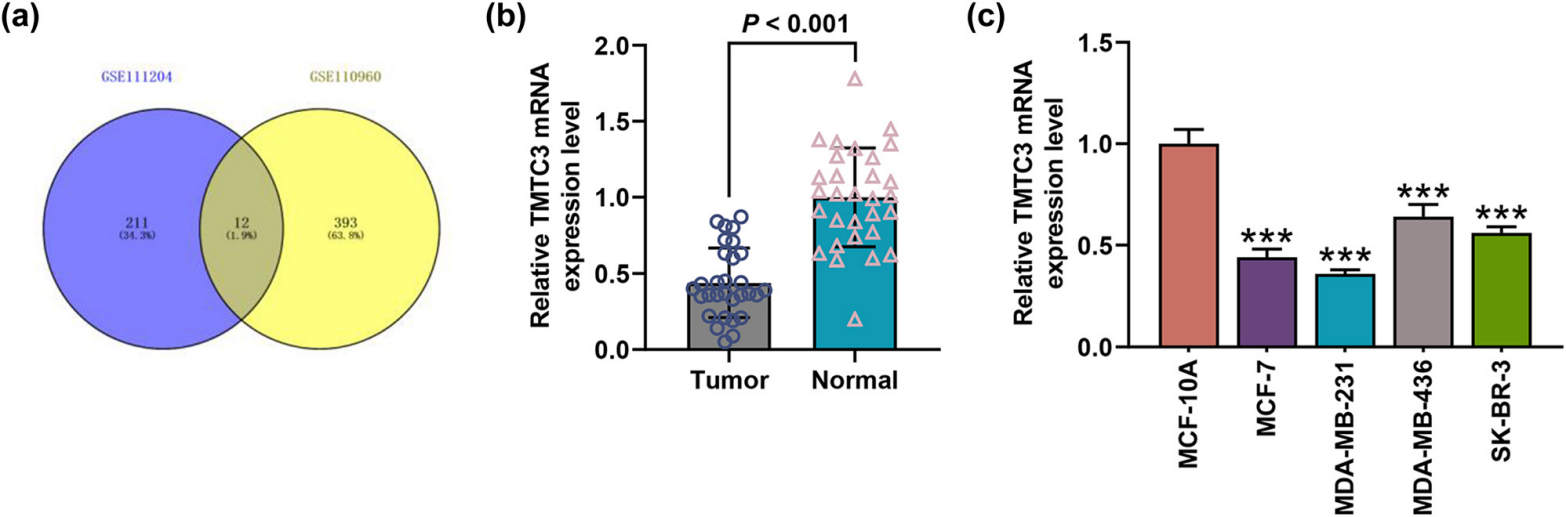
TMTC3 was lowly expressed in BC tissue and cell. (a) Dataset GSE110960 (effect of restricted glycolysis on gene expression in MCF-7 cells) and GSE111204 (effect of restricted glycolysis on gene expression in MDA-MB-231 cells) were used to sort the candidate gene for our study, which was available from GEO (https://www.ncbi.nlm.nih.gov/geo/). A Venn diagram was drawn based on the results. (b) Relative TMTC3 expression in BC (tumor) and adjacent (normal) tissue was calculated via reverse-transcription quantitative PCR (*n* = 30 for each group). GAPDH was used as the internal control. (c) Relative TMTC3 expression in BC cells (MCF-7, MDA-MB-231, MDA-MB-436, and SK-BR-3) and breast epithelial cell MCF-10A was quantified with reverse-transcription quantitative PCR. GAPDH was used as the internal control. All data were expressed as mean ± SD, which was indicative of three independent tests. ^***^ *p <* 0.001, vs MCF-10A. TMTC3: transmembrane O-mannosyltransferase targeting cadherins 3; BC: breast cancer.

To sort the candidate gene for our study, we first downloaded the datasets of GSE110960 (effect of restricted glycolysis on gene expression in MCF-7 cells) and GSE111204 (effect of restricted glycolysis on gene expression in MDA-MB-231 cells) from GEO. A Venn diagram was subsequently drawn to sort the common DEGs, 12 of which were then identified, including TMTC3 (Figure 1a). TMTC3 contributes to the *O*-mannosylation of E-cadherin, and dysregulation of glycosylation is one of the important mechanisms leading to tumor heterogeneity; in addition, TMTC3 is involved in the regulation of ERS, and ERS affected the malignant growth and progression of tumors. Thus, TMTC3 was used for our studies.

Then we measured TMTC3 expression in both BC and adjacent tissue, and a low TMTC3 expression was evidenced in BC tissue (Figure 1b, *p* < 0.001). Likewise, we also detected the expression of TMTC3 in BC cells and breast epithelial cell MCF-10A, and the result showed that TMTC3 expression was lower in BC cells (MCF-7, MDA-MB-231, MDA-MB-436, SK-BR-3) than that in MCF-10A cells (Figure 1c, *p* < 0.001). Among these BC cells, TMTC3 expression in MCF-7 and MDA-MB-231 cells was lower than other BC cells, and thus these two cells were used for our subsequent studies.

### 3.2 Glycolysis promotion represses TMTC3 expression yet enhances lactic acid production and growth of BC cell, while inhibition elicited contrary results

**Figure 2:**
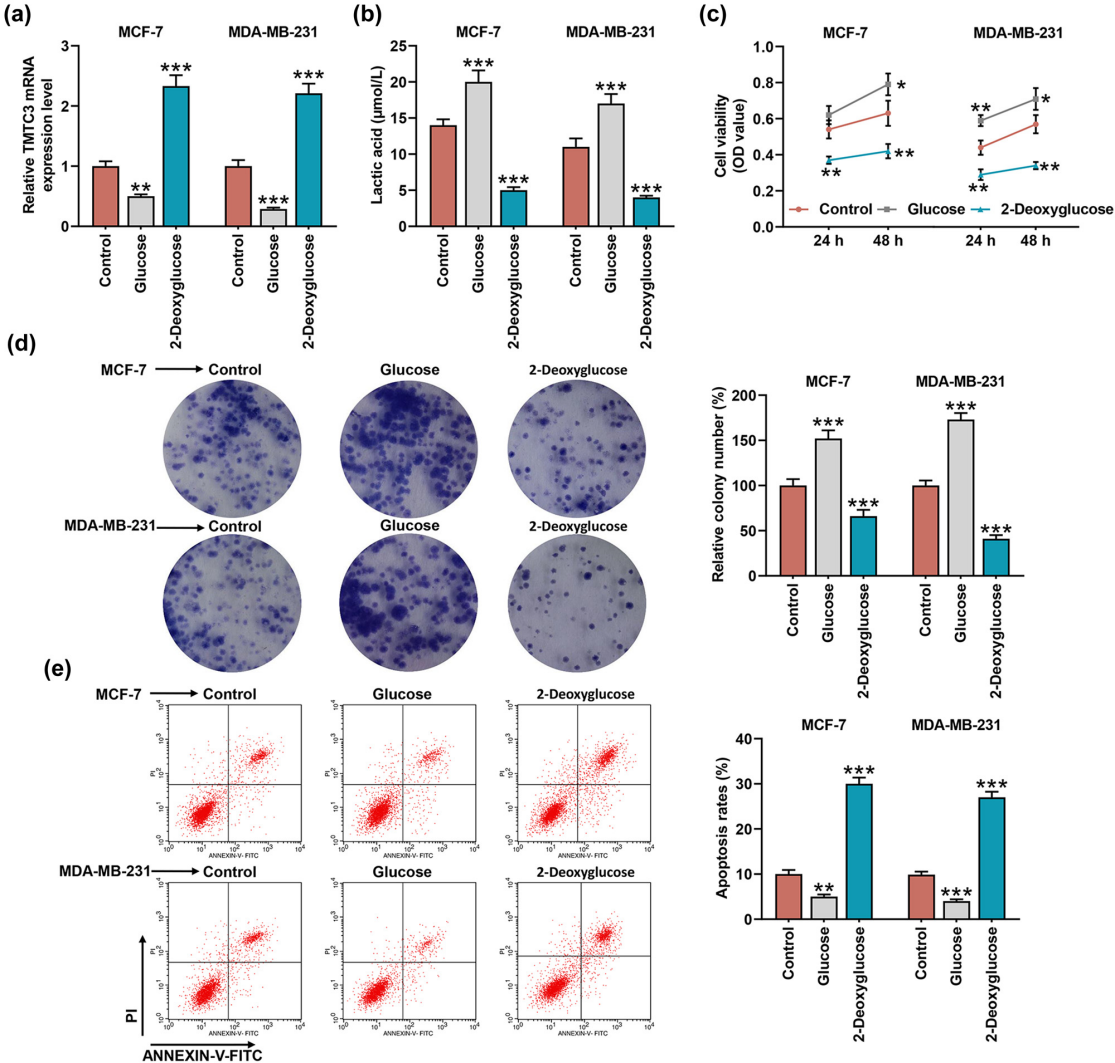
Glycolysis promotion represses TMTC3 expression yet enhances the lactic acid production and growth of BC cell, while the inhibition did contrarily. (a) The effects of glucose (which promoted glycolysis) and 2-deoxyglucose (which repressed glycolysis) on TMTC3 expression in BC cells MCF-7 and MDA-MB-231 were confirmed with reverse-transcription quantitative PCR. GAPDH was used as internal control. (b–e) The effects of glucose and 2-deoxyglucose on lactic acid production (b), viability (c), proliferation (d), and apoptosis (e) of BC cells MCF-7 and MDA-MB-231 were evaluated via lactic acid production, CCK-8, colony formation and flow cytometry assays, respectively. All data were expressed as mean ± SD, which was indicative of three independent tests. ^*^ *p <* 0.05, ^**^ *p <* 0.01, ^***^ *p <* 0.001, vs control. OD: optical density.

For subsequent procedures, we cultured both MCF-7 and MDA-MB-231 cells in their respective media supplemented with glycose or 2-deoxyglucose, which have been suggested to promote or repress glycolysis [24]. In the beginning, we measured TMTC3 expression in BC cells after the cells were cultured in the media supplemented with glycose or 2-deoxyglucose, and it was found that the expression of TMTC3 was repressed following the supplementation of glycose, whereas 2-deoxyglucose supplementation elevated TMTC3 expression in BC cells (Figure 2a, *p* < 0.01).

Lactic acid was one of the end-products of aerobic glycolysis, which had both the capability to change the tumor microenvironment and an impact on cancer-associated cells [28]. It was also demonstrated that after the supplementation of glycose, the concentration of lactic acid in BC cells was evidently raised, while the additional supplementation of 2-deoxyglucose reduced the concentration of lactic acid (Figure 2b, *p* < 0.001). When it comes to viability (Figure 2c), proliferation (Figure 2d), and apoptosis (Figure 2e), it was discovered that the supplementation of glycose raised the viability and colony formation rate yet reduced apoptosis in BC cells (Figure 2c–e, *P* < 0.05); however, contrary results were displayed in the BC cells when the culture medium was added with 2-deoxyglucose. That is, the supplementation of 2-deoxyglucose decreased the viability at 24 and 48 h and colony formation rate yet increased apoptosis in BC cells (Figure 2c–e, *p* < 0.01). The aforementioned results suggested that promoting glycolysis using glucose represses TMTC3 expression yet enhances lactic acid production and growth in BC cells, whereas inhibiting glycolysis via 2-deoxyglucose increased TMTC3 expression yet repressed the growth of BC cells.

**Figure 3:**
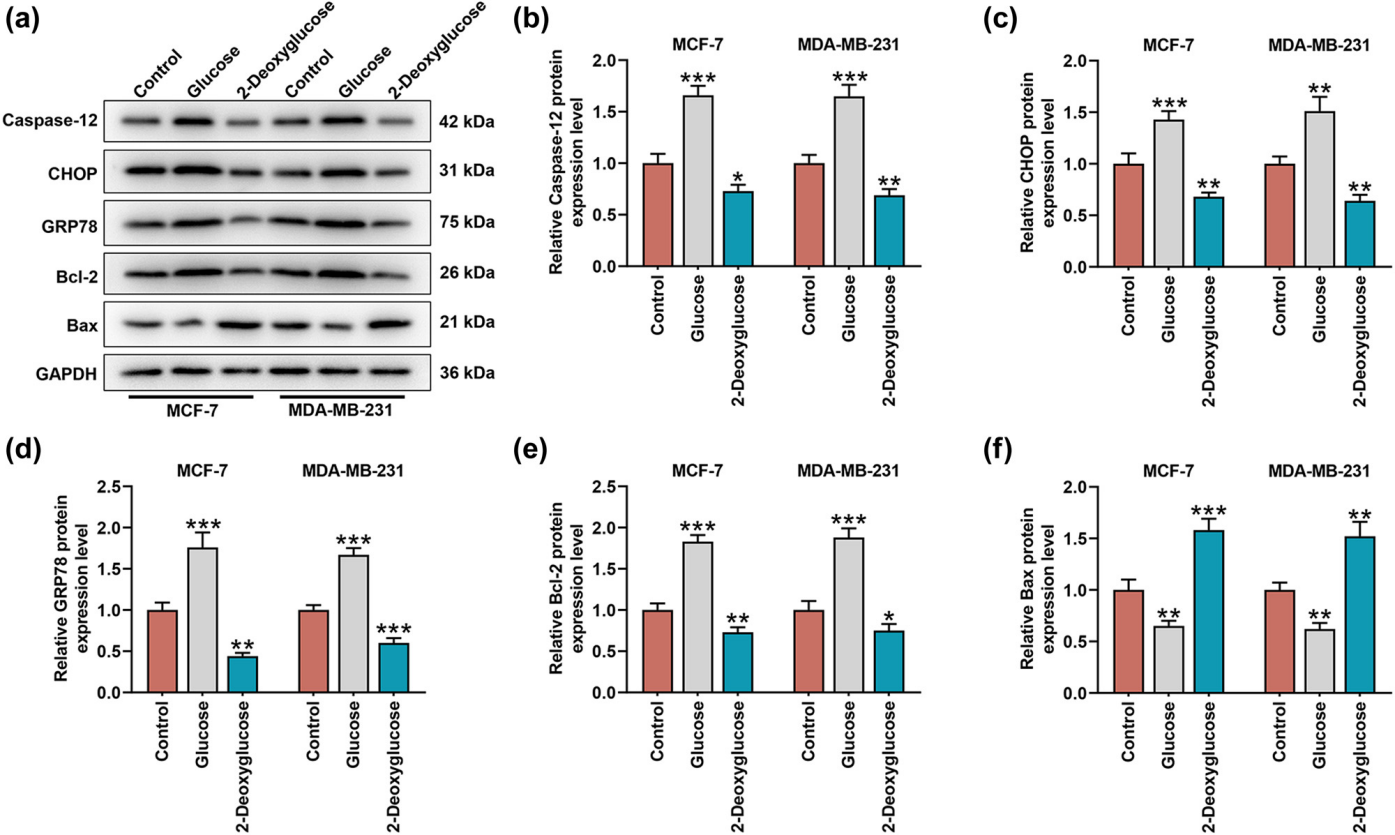
Glycolysis posed a regulatory effect on the expressions of factors related to both ER stress and apoptosis in BC cell. (a–f) The effects of glucose and 2-deoxyglucose on ER stress- and apoptosis-related factors (Caspase-12 (b), CHOP (c), GRP78 (d), Bcl-2 (e), and Bax (f)) in BC cells MCF-7 and MDA-MB-231 were unveiled with Western blot. GAPDH was used as internal control. All data were expressed as mean ± SD, which was indicative of three independent tests. ^*^ *p <* 0.05, ^**^ *p <* 0.01, ^***^ *p <* 0.001, vs control. ER: endoplasmic reticulum; CHOP: C/EBP homologous protein; GRP78: glucose-regulated protein 78; Bax: Bcl-2 associated X; Bcl-2: B-cell lymphoma-2.

To additionally confirm the mechanisms, we measured the expressions of both ER stress- and apoptosis-related factors in BC cells, as evidenced by the interaction among ER stress, apoptosis, and glycolysis [29,30]. All ER stress-related factors, including Caspase-12, CHOP, and GRP78, and apoptosis-related factor Bcl-2 were promoted but another apoptosis-related factor Bax was suppressed in BC cells cultured in the medium with glucose (Figure 3a–f, *p* < 0.01). However, in those cells maintained in the medium with 2-deoxyglucose, the decreased levels on ER stress-related factors, including Caspase-12, CHOP, and GRP78, and apoptosis-related factor Bcl-2 were exhibited yet that of apoptosis-related factor Bax was increased (Figure 3a–f, *p* < 0.05).

### 3.3 Overexpressed TMTC3 abrogated the effects of glycolysis in BC cells

**Figure 4:**
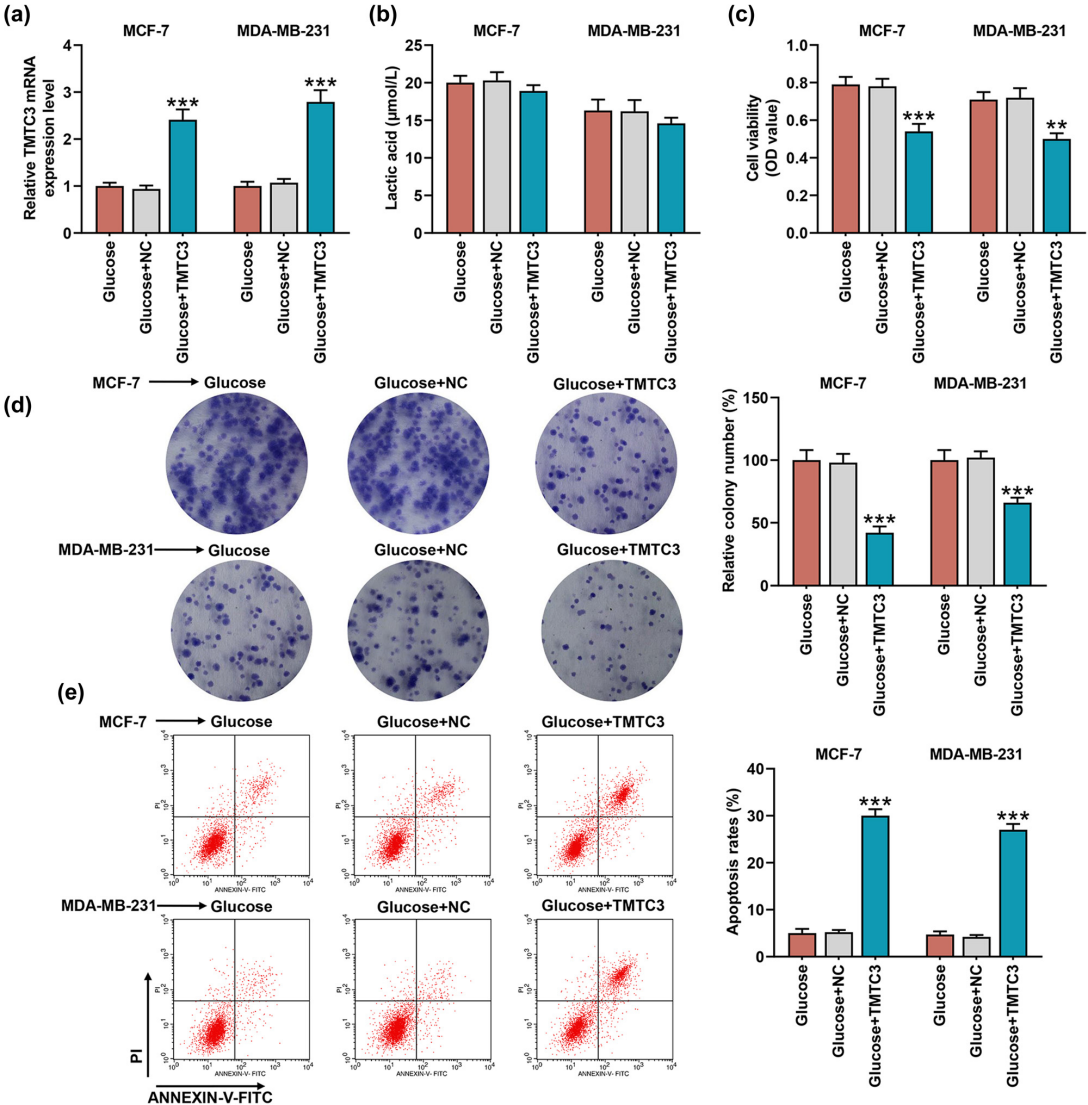
Overexpressed TMTC3 abrogated the effects of glycolysis. (a) The effects of glucose and TMTC3 on TMTC3 expression in BC cells MCF-7 and MDA-MB-231 were unveiled in accordance with the results of reverse-transcription quantitative PCR. GAPDH was used as internal control. (b–e) The effects of glucose and TMTC3 on the lactic acid production (b), viability (c), proliferation (d), and apoptosis (e) of BC cells MCF-7 and MDA-MB-231 were evaluated via lactic acid production, CCK-8, colony formation and flow cytometry assays, respectively. All data were expressed as mean ± SD, which was indicative of three independent tests. ^**^ *p <* 0.01, ^***^ *p <* 0.001, vs glucose + NC. NC: negative control.

To further confirm the interaction between glycolysis and TMTC3 in BC cells, we transfected TMTC3 overexpression plasmid into BC cells, which were subsequently cultured in the medium supplemented with glycose. The successful transfection was evidenced by the increased TMTC3 expression in BC cells which have been maintained in the medium with glycose (Figure 4a, *p* < 0.001). No evident effect on the lactic acid concentration in BC cells was displayed in BC cells following the transfection of TMTC3 overexpression plasmid (Figure 4b), whereas it was clear that TMTC3 overexpression decreased the viability and colony formation yet enhanced the apoptosis of BC cells cultured with glycose (Figure 4c–e, *p* < 0.01). Herein, we concluded that overexpressed TMTC3 could have abrogated the effects of glycolysis promotion on the malignant behaviors of BC cells.

**Figure 5:**
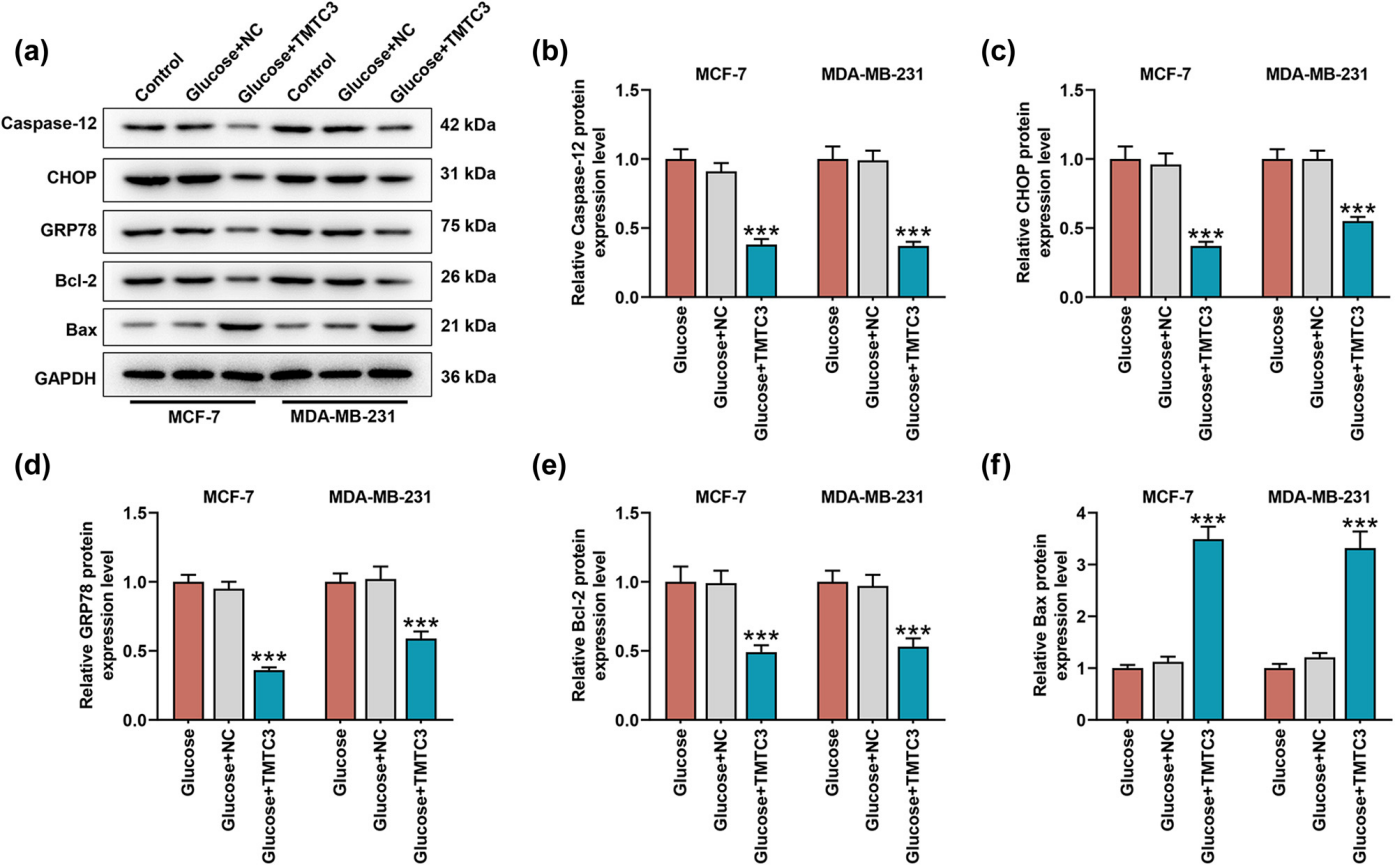
Both TMTC3 and glycolysis posed regulatory effects on the expressions of both ER stress- and apoptosis-related factors in BC cell. (a–f) The effects of glucose and TMTC3 on ER stress- and apoptosis-related factors (Caspase-12 (b), CHOP (c), GRP78 (d), Bcl-2 (e), and Bax (f)) in BC cells MCF-7 and MDA-MB-231 were unveiled with Western blot. GAPDH was used as internal control. All data were expressed as mean ± SD, which was indicative of three independent tests. ^***^ *p <* 0.001, vs glucose + NC.

As for the ER stress- and apoptosis-related factors, TMTC3 overexpression diminished the effects of glycose on promoting Caspase-12, CHOP, GRP78, and Bcl-2 yet repressing Bax in BC cells (Figure 5a–f, *p* < 0.001). The results thus suggested that TMTC3 overexpression reversed the regulatory effects of glycolysis on the ER stress- and apoptosis-related factors in BC cells.

## 4 Discussion

In this study, we investigated the effects of glycolysis and TMTC3 in BC. We found that glycolysis promoted the proliferation and ER stress but inhibited the apoptosis of BC cells by inhibiting TMTC3. This finding enriches the research on the mechanism of glycolysis in BC and provides a new therapeutic target for BC-targeted therapy.

Recent years have witnessed the emergence of cellular metabolism as a major biological node which governs the cellular behavior; multiple molecular mechanisms, both intrinsic and extrinsic, have been suggested to converge to alter the core cellular metabolism and provide support for the basic needs used for the division of cells [31,32]. The critical determinant role of cellular metabolism in the viability and function of cancer cells has been additionally addressed, and tumorigenesis is dependent on the reprogramming of cellular metabolism as the consequence of direct and indirect oncogenic mutations [33,34]. Different from the normal differentiated cells, which are primarily dependent on mitochondrial oxidative phosphorylation for the generation of the energy required for cellular process, cancer cells, instead, rely on the aerobic glycolysis [35]. For a long time, glycolysis has been seen as the major metabolic process for energy production and anabolic growth in cancer cells, and glycolytic inhibition in cancer cells has been considered as a novel strategy for overcoming the drug resistance related to mitochondrial respiratory defect and hypoxia [36,37]. Therefore, it is of great value to gain further and better understanding on the molecular mechanism of aerobic glycolysis so as to identify and recognize novel therapeutic targets for cancer [25]. The GRP78-mediated glycolysis in BC is shown to be targeted by betulinic acid, which represses the metastasis of BC, for instance [13]. In our current study, we also proposed that the inhibition of glycolysis with 2-deoxyglucose was associated with lactic acid production and repressed growth of BC cells, providing another evidence on the interaction between the inhibition of glycolysis and the repressive growth of BC cells.

Increasing evidence has also highlighted the regulation of aerobic glycolysis in the process of carcinogenesis, lactate production for carcinogenesis could be the propose and explanation for Warburg Effect [38,39]. As the end-product of aerobic glycolysis formed and utilized under the fully aerobic conditions, lactate is the fuel source for cancer cells to promote inflammation, angiogenesis, metastasis, immune evasion, etc. [40]. Meanwhile, the fundamental role of aerobic glycolysis in supporting cell growth and the use of aerobic glycolysis during rapid proliferation have been addressed in many cells at the same time, where aerobic glycolysis links the high glucose fermentation rate to the unrestrained proliferation and progression of cancer cells [41,42]. Glycolysis, additionally, can provide the “building blocks” for the macromolecule synthesis of proliferated cancer cells, including carbon skeletons, nicotinamide adenine dinucleotide phosphate, and adenosine triphosphate, which, in turn, rewires the metabolic pathway for the survival and growth of cancer cells [43]. Associated with the high glucose intake and lactate generation rate, glycolysis prevents the apoptosis of cancer cells due to the regulation of cellular metabolites on the function of pro- or anti-apoptotic proteins, which, in turn, control the metabolism via the limitation of glycolysis [30,44]. As one of the major protein families implicated in the regulation of cell death and aberrantly expressed in cancers, the Bcl-2 family of proteins is one of the critical mediators for the metabolic pathways [45]. The balance on the Bcl-2 family of proteins via glucose metabolism may be a pivotal aspect regarding how aerobic glycolysis leaves an effect on cell fate, providing evidence on the importance of the metabolic shift to the survival of cancer cells [45,46]. Several proteins from Bcl-2 family proteins, such as Mcl-1, NOXA, and Bad, have been shown to be involved in the reprogrammed metabolism in cancer cells [45]. In our current study, we also evidenced the interaction between glycolysis and the apoptosis of BC cells. Specifically, the promotion of glycolysis inhibits the apoptosis of BC cells, with the increased level of Bcl-2 yet the decreased expression of Bax, whereas the inhibition of aerobic glycolysis did conversely.

Furthermore, as a hallmark in multiple solid malignancies, ER stress, as well as its associated unfolded protein response, initiates multiple survival mechanisms in cancer cells, and a prior discovery has evidenced the implication of ER stress in Chromium (Cr)(vi)-induced glycolysis in lung carcinoma cell A549, where phenylbutyric acid, the inhibitor of ER stress, unleashes a repressive effect on Cr(vi)-induced glycolysis [47]. It is also shown that the administration of 2-deoxyglucose is associated with decreased cell viability and increased ER stress in neuroblastoma cells, with the upregulation on both ER molecular chaperone GRP78 and the pro-death protein CHOP [48]. A contrary result, however, was confirmed in our study, from which we discovered that the promotion of glycolysis using glucose enhanced the ER stress in BC cells, along with the upregulated levels of CHOP, GRP78, and Caspase-12 (a central caspase implicated in the ER stress-mediated apoptosis [49]), providing another evidence concerning the participation of ER stress in the progression of BC.

In accordance with the previous publications, the TMTC proteins are those located in the ER and predicted to be implicated in both calcium ion (Ca^2+^) regulation and protein folding [22,50]. Meanwhile, the biallelic TMTC3 mutations result in cobblestone lissencephaly, intellectual disability, and epilepsy, in addition to the highlight on its possible implication in BC [23,51,52]. In our current study, we not only reconfirmed the role of TMTC3 played in BC but also provided evidence regarding the possible interaction between TMTC3 and glycolysis. In other words, in addition to the confirmation suggesting the low expression of TMTC3 in BC, overexpressed TMTC3 abrogated the effects of glucose on promoting the growth of BC cells, along with the downregulation on glucose-induced increased Caspase-12, GRP78, and Bcl-2 expressions yet decreased Bax expression, thus emphasizing both the implication of TMTC3 and the interaction between TMTC3 and glycolysis in BC cells.

It should be noted that despite the confirmation on the implication of TMTC3 and the interaction between TMTC3 and glycolysis in BC, all results were concluded based on the experiments *in vitro*. The effects of TMTC3 *in vivo* were lack of an equivalent verification, which is one of the major shortcomings of our current study, making further validating studies required to complete the results of our study. In addition, whether glycolysis can affect the development of BC by regulating other genes needs to be a more comprehensive and in-depth study.

## 5 Conclusion

Taken together, we have provided another evidence concerning the implication of glycolysis in BC. Specifically, we confirm that the inhibition of glycolysis is associated with the repressed viability and proliferation but enhanced apoptosis in BC cells. In addition, we not only recognize the implication of TMTC3, a member of ER transmembrane *O*-mannosyltransferase, in BC, but also unveil the interaction between TMTC3 and glycolysis in BC. In addition, the overexpression of TMTC3 abolishes the effects of glucose on the growth and ER stress of BC cells. We hope the result from our current study can be used as the theoretical basis for the research on the possible participation of glycolysis and TMTC3 in BC, with a potentially viable therapeutic method for BC.
